# Author Correction: Record sea surface temperature jump in 2023–2024 unlikely but not unexpected

**DOI:** 10.1038/s41586-025-08923-1

**Published:** 2025-04-04

**Authors:** Jens Terhaar, Friedrich A. Burger, Linus Vogt, Thomas L. Frölicher, Thomas F. Stocker

**Affiliations:** 1 Climate and Environmental Physics, Physics Institute, University of Bern, Bern, Switzerland; 2 Oeschger Center for Climate Change Research, University of Bern, Bern, Switzerland; 3 LOCEAN/IPSL, Sorbonne Université, CNRS, IRD, MNHN, Paris, France

**Keywords:** Climate and Earth system modelling, Attribution, Physical oceanography

Correction to: *Nature* 10.1038/s41586-025-08674-z Published online 12 March 2025

In the version of the article initially published, the date range shown in green in Extended Data Fig. 4g was shown as 2041–2050 instead of 2042–2051. Similarly, the colour keys in Extended Data Figs. 1l and 4l were duplicates of those in Extended Data Figs. 1k and 4k, respectively, and have now been increased by two years for the time spans in each key. Finally, the title of Extended Data Fig. 4 has been updated to read “The gap in global sea surface temperatures created by the simulated record-shattering jumps is closed in most simulations in the years following the jump.” The figures and text have been corrected in the HTML and PDF versions of the article.

